# Optimized Viability-ddPCR with Triton X-100 Enhancement for Selective Detection of Live *Salmonella enterica* and *Cronobacter sakazakii*

**DOI:** 10.4014/jmb.2508.08007

**Published:** 2026-01-13

**Authors:** Minkyu Park, Changwoo Park, Seung Bum Kim, Seil Kim

**Affiliations:** 1 Biometrology Group, Korea Research Institute of Standards and Science (KRISS), Daejeon 34113, Republic of Korea; 2 Department of Microbiology and Molecular Biology, College of Bioscience and Biotechnology, Chungnam National University, Daejeon 34134, Republic of Korea; 3 Department of Precision Measurement, University of Science & Technology (UST), Daejeon 34113, Republic of Korea

**Keywords:** Foodborne diseases, Real-time polymerase chain reaction, Droplet digital PCR, PMA, Triton X-100

## Abstract

Viability PCR (v-PCR) was optimized using propidium monoazide (PMA) and Triton X-100 for the selective detection of live foodborne pathogens in this study. The colony-forming unit (CFU) method, conventionally used for detecting live foodborne pathogens, is time-consuming. Quantitative PCR (qPCR) and droplet digital PCR (ddPCR) have emerged as rapid alternatives for pathogen detection, which provide accurate detection at low bacterial concentrations. However, these methods cannot distinguish between live and dead bacteria. We investigated the optimization of v-PCR conditions using PMA concentrations (10-200 μM) and Triton X-100 concentrations (0-1%) for *Salmonella enterica* subsp. *enterica* and *Cronobacter sakazakii*. PMA at 100 μM effectively inhibited the amplification of dead bacteria, whereas 1% Triton X-100 enhanced PMA efficiency. The optimized v-ddPCR method successfully distinguished live from dead bacteria, although discrepancies were observed between CFU counts and ddPCR copy numbers. Triton X-100 treatment reduced these discrepancies, bringing the ddPCR copy numbers closer to CFU counts obtained from traditional culture methods. This optimized v-ddPCR protocol provides a rapid and reliable approach for detecting viable foodborne pathogens in food safety applications, that offers enhanced sensitivity compared with conventional culture-based methods.

## Introduction

Foodborne illnesses are among the oldest known diseases that affect humanity [1]. These illnesses commonly occur through contamination of food by microbial or chemical sources and can lead to significant public health problems [2]. Common symptoms of foodborne illnesses include digestive issues such as vomiting and diarrhea [1, 3]. In Korea, foodborne illness surveillance data from 1996 to 2023 shows significant fluctuations over time, with a cumulative total of 6,001 outbreaks involving 150,063 patients recorded during this 28-year period [4]. According to the MFDS (Ministry of Food and Drug Safety.), both *Salmonella* spp. and *Cronobacter* spp. are pathogens that cause foodborne disease outbreaks [5, 6]. Both *Salmonella* and *Cronobacter* are rod-shaped, gram-negative bacteria. *Salmonella* spp. is one of the most common causes of foodborne illnesses [3, 7-10]. In contrast, the rate of foodborne illnesses caused by *Cronobacter* spp. is low. However, because it is associated with high mortality rates, the risk is comparable to that of *Salmonella* spp. [3, 9, 11]. Despite stringent food safety measures, outbreaks continue to occur annually. Since foodborne illnesses occur only in living bacteria, the accurate detection of viable cells is crucial. For this study, *Salmonella enterica* subsp. *enterica* strain LT2:DSM 17058 and *Cronobacter sakazakii* strain KCTC 2949 were selected as representative foodborne pathogens. *S. enterica* LT2:DSM 17058 is a well-characterized reference strain widely used in food safety research, while *C. sakazakii* KCTC 2949 represents a clinically relevant strain associated with foodborne illness outbreaks.

The conventional method for detecting pathogenic bacteria involves culturing on agar plates to count the colony-forming units (CFU). However, this method has internal limitations, including low sample concentrations, slow bacterial growth rates, and complex culture conditions, as well as external limitations, such as personnel constraints and financial resources [12]. Owing to these limitations, several alternative approaches for bacterial detection have been developed. For example, quantitative polymerase chain reaction (qPCR) has been widely adopted as a major alternative detection method [13-15]. Several studies have also used droplet digital polymerase chain reaction (ddPCR) [12, 14, 15]. qPCR, also known as real-time PCR, has become one of the most widely used experimental techniques in modern molecular biology [13, 16, 17]. This method offers advantages, such as real-time monitoring of amplification, reduced cross-contamination risk, and the ability to distinguish between specific and non-specific amplification [12,13,17]. In contrast, ddPCR has not been designated as an official detection method by the MFDS. However, ddPCR employs a fluorescence-based detection system similar to that used for qPCR, which could enhance its applicability [18-20]. Approximately 20,000 droplets were generated in ddPCR by partitioning the samples and fluorescence from the successfully amplified target nucleic acids was detected [21, 22]. By counting fluorescent droplets rather than measuring fluorescence intensity, ddPCR provides a more sensitive, accurate, and absolute quantification than qPCR in low-concentration samples [22-24]. ddPCR is particularly valuable for detecting trace amounts of foodborne pathogens in environmental samples [25-27].

Both qPCR and ddPCR provide highly sensitive and accurate pathogen detection [23, 28, 29]. However, both methods are unable to distinguish live bacteria from dead bacteria. To address this limitation, viability PCR (v-PCR) has been developed. v-PCR can be used to distinguish between live and dead bacteria by only detecting the former [30]. It uses photoreactive dyes, such as ethidium monoazide (EMA) and propidium monoazide (PMA) [26, 28, 29]. These dyes penetrate the membranes of dead bacteria and selectively bind their genomic DNA (gDNA), thereby inhibiting PCR amplification [28, 30, 31]. In contrast, intact membranes of live bacteria prevent PMA penetration and thus only live bacterial gDNA is amplified [29]. EMA is dissolved in dimethyl sulfoxide (DMSO), which can damage live bacterial membranes, whereas PMA is dissolved in nuclease-free water without affecting live bacteria [26, 28, 30]. PMA is therefore preferred because it rarely damages the membranes of live bacteria and penetrates dead bacteria more selectively than EMA does. This method allows for selective amplification of gDNA from live bacteria, and thus enables a clear comparison between live and dead bacteria [23, 28, 31]. However, surfactants can enhance the efficiency of PMA penetration into already compromised dead bacterial membranes at concentrations below the threshold and significantly affecting intact live bacterial membranes [32]. This concentration-dependent effect allows for improved selectivity for PMA binding. Surfactants are classified into cationic, anionic, zwitterionic, and nonionic types [33]. Among these, nonionic surfactants are favored owing to their low cost, simplicity, and low toxicity [33, 34]. Triton X-100 is a commonly used water-soluble non-ionic agent [33]. The optimal concentration of Triton X-100 was evaluated to minimize bacterial damage and enhance the penetration efficiency of PMA [32, 35,36].

This study evaluated detection methods that specifically target foodborne pathogens such as *S. enterica* and *C. sakazakii*. Both bacteria are particularly concerning in terms of food safety because of their severe health implications [1, 3, 8, 10]. This study used PMA to optimize conditions using qPCR with *S. enterica*, and subsequently verified the optimized conditions using ddPCR with *S. enterica* and *C. sakazakii*. Triton X-100 was incorporated into the v-PCR to enhance the selective detection of live bacteria. As only live bacteria pose potential safety concerns, their detection is especially important for assessing contamination risks in post-sterilization and processed food products. By integrating these complementary detection methods, the suggested approach is expected to provide rapid, sensitive, and accurate quantification of live foodborne pathogens, which is particularly valuable for ensuring the safety of food products.

## Materials and Methods

### Bacterial Strains and Culture Conditions

*Salmonella enterica* subsp. *enterica* strain LT2:DSM17058 was obtained from DSMZ (Deutsche Sammlung von Mikroorganismen und Zellkulturen, Germany); *Cronobacter sakazakii* strain KCTC2949 was obtained from KCTC (Korean Collection for Type Cultures, Jeongeup, Republic of Korea). Both strains have been extensively validated using PCR-based detection methods [37]. For liquid culture, both bacteria were grown in a Brain Heart Infusion (BD Biosciences, USA) at 37°C with shaking at 200 rpm for 16 h. For plate cultures, bacteria were grown on BHI agar at 37°C for 16 h. Single colonies were inoculated into BHI broth and the optical density (OD_600_) was adjusted to 1.0.

### Enumeration Using Colony-Forming Unit (CFU)

Following the Korean Food Code of the MFDS [37], bacterial suspensions were serially diluted to obtain the recommended range of 15-300 CFU/plate. The samples were spread onto BHI agar and incubated at 37°C for 16 h. The colony counts were converted to CFU/ml using Eq. (1) below:


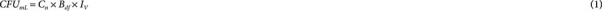


The *CFU_mL_* represents the colony forming units per ml, *C_n_* is the colony numbers counted on plates, *B_df_* denotes the dilution factor applied for plating, and *I_V_* is the inoculated volume factor, and the results are expressed as CFU per ml of original bacterial culture.

### Sample Preparation

**Fig. 1:**
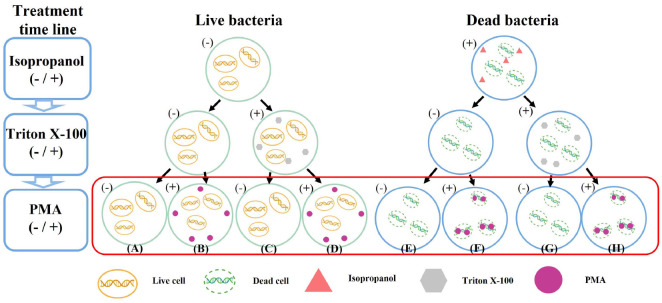
Bacterial experimental conditions. The minus (-) and plus (+) symbols indicate untreated and treated conditions. (**A**) Live bacteria (- PMA, - TX), (**B**) Live bacteria (+ PMA, - TX), (**C**) Live bacteria (- PMA, + TX), (**D**) Live bacteria (+ PMA, + TX), (**E**) Dead bacteria (- PMA, - TX), (**F**) Dead bacteria (+ PMA, - TX), (**G**) Dead bacteria (- PMA, + TX), (**H**) Dead bacteria (+ PMA, + TX).

The optical density of bacterial cultures was measured using a spectrophotometer (Lambda 25 UV/Vis spectrometer, PerkinElmer, USA) and adjusted to an OD_600_ of 1.0 by dilution. To prepare dead bacteria, live bacteria were treated with 70% isopropanol for 10 min at room temperature [38, 39], followed by centrifugation at 12,000 × *g* for 5 min and resuspension in BHI broth. Subsequently, Both live and dead bacterial samples were divided into Triton X-100 treated and untreated groups; the former was treated with 1% Triton X-100 for 10 min at room temperature followed by centrifugation at 12,000 × *g* for 10 min. The pellets were then resuspended in BHI broth. The samples were separated into the PMA-treated and untreated groups (Fig. 1). For the PMA-treated samples, PMA Enhancer (PMA Enhancer for Gram-negative bacteria, 5X Solution, Biotium, USA) and PMAxx Dye (PerkinElmer, USA) were added to reach final concentrations of 10, 50, 100, and 200 μM. An appropriate volume of nuclease-free water was added to the untreated samples to equalize the volume of the PMA-treated samples. Both groups were incubated in the dark at room temperature for 15 min, followed by exposure to blue light (465-475 nm) for 15 min using a PMA-Lite LED photolysis device (Biotium) before gDNA extraction. gDNA was extracted using a GenElute Bacterial Genomic DNA Kit (Sigma-Aldrich, USA), which employs a silica-based membrane for DNA purification according to the manufacturer's instructions. To standardize bacterial concentrations for consistent v-PCR performance, preliminary experiments were conducted to optimize OD_600_ values. Three conditions were evaluated: an averaged OD_600_ of 0.854 (derived from the mean values of initial bacterial culture measurements), a recommended OD_600_ of 1.0 (based on previous viability-PCR studies), and a higher OD_600_ of 2.0 (for a comparative analysis of concentration effects on PMA binding efficiency).

### Assessment of PMA Inhibitory Effect on Isolated Genomic DNA

gDNA was extracted from live and dead *S. enterica* and *C. sakazakii* without PMA treatment. The extracted gDNA samples were divided into PMA-untreated and directly PMA-treated groups. An equivalent volume of nuclease-free water was added to PMA-untreated cells. For the PMA-treated groups, PMA and PMA enhancers were added at a final concentration of 100 μM. All samples were incubated in the dark at room temperature for 15 min, followed by 15 min of exposure to blue light using a PMA-Lite LED Photolysis Device. The samples were then stored at 4°C for subsequent experiments.

### Primer and Probe Specifications

Primer and probe sets were recommended by the MFDS Food Code [37] in Korea, and all primer and probe sets were synthesized by Macrogen (Macrogen, Republic of Korea). The target gene of *S. enterica* is invasion protein A (*InvA*). While *C. sakazakii* had no designated target gene specified in the MFDS Food Code, this primer set targeted a non-coding intergenic region between the *rpsU* and *dnaG* genes. The detailed sequences for both *S. enterica* and *C. sakazakii* primer and probe sets are provided in Table 1.

### Quantitative PCR (qPCR)

qPCR was performed using TaKaRa Probe qPCR Mix (Takara Bio, Japan). The reaction mixture was prepared in a total volume of 20 μl, which included 10 μl of qPCR Mix (2X), 0.4 μl of forward primer (10 μM), 0.4 μl of reverse primer (10 μM), 0.8 μl of probe (5 μM), 0.4 μl of Rox Reference Dye (50X), 6 μl of nuclease-free water, and 2 μl of gDNA. The qPCR conditions were as follows: initial denaturation at 95°C for 20 sec, followed by 40 cycles of 95°C for 1 sec and 60°C for 20 sec using the StepOnePlus Real-Time PCR System (Applied Biosystems, USA).

### Droplet Digital PCR (ddPCR)

ddPCR Supermix for Probes (Bio-Rad Laboratories, USA) was used for ddPCR amplification. The same primer and probe sets as those described for qPCR were used. The reaction mixture was prepared in a total volume of 20 μl, which included 10 μl of 2X ddPCR Supermix for Probes, 1 μl of forward primer (10 μM), 1 μl of reverse primer (10 μM), 1 μl of probe (5 μM), 2 μl of nuclease-free water, and 5 μl of gDNA. The ddPCR conditions consisted of initial denaturation at 95°C for 10 min, followed by 40 cycles at 94°C for 30 sec, 60°C for 1 min, and one cycle at 98°C for 10 min. To prevent individual partitions of the ddPCR droplets from breaking, the ramp rate of the PCR instrument (Verity 96-well Thermal Cycler, Thermo Fisher Scientific, USA) was set at 20% (approximately 0.8°C/s). The copy numbers were converted to copies/ml using Eq. (2):


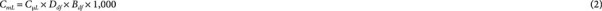


*C_mL_* represents the normalized copy number per ml, *C*_μ*L*_ is the copy number measured by ddPCR (copies/μl), *D_df_* denotes the dilution factor applied for ddPCR template preparation, *B_df_* is the broth dilution factor to normalize back to the original bacterial culture concentration, and 1,000 is the conversion factor from μl to ml.

Copy numbers were back-calculated to represent the original bacterial culture concentration by applying the broth dilution factor, allowing accurate comparison with CFU enumeration performed on the same original culture.

### Statistical Analysis

Statistical comparisons were performed using independent t-tests to evaluate the differences in qPCR Ct values between experimental conditions. For the PMA concentration experiments, all conditions (live (-PMA), live (+PMA), and dead (-PMA)) were compared against dead (+PMA) as the reference group at each concentration (10, 50, 100, and 200 μM). For the Triton X-100 concentration experiments, the conditions were similar to those of dead (+PMA) as the reference at each concentration (0%, 0.25%, 0.5%, and 1%). Each comparison included three biological replicates for each condition. Statistical significance was defined as *P* < 0.05. Statistical analyses were performed using the Python scipy.stats package.

## Results

### *S. enterica* and *C. sakazakii* CFU Counts

**Fig. 2:**
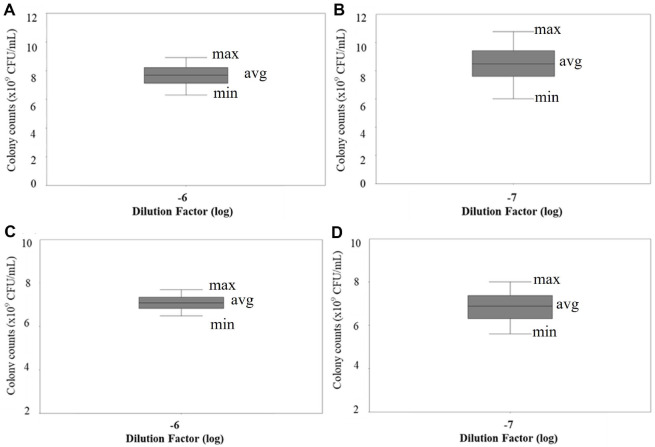
Colony-forming unit (CFU) counts at different dilution factors (log). Average (avg), maximum (max), and minimum (min) colony counts are shown. (**A**) *S. enterica* at -6 log dilution; Avg is 7.72 × 10^9^ CFU/ml, max is 8.92 × 10^9^ CFU/ml and min is 6.28 × 10^9^ CFU/ml. (**B**) *S. enterica* at -7 log dilution; Avg is 8.4 × 10^9^ CFU/ml, max is 10.0 × 10^9^ CFU/ml and min is 6.0 × 10^9^ CFU/ml. (**C**) *C. sakazakii* at -6 log dilution; Avg is 7.08 × 10^9^ CFU/ml, max is 7.68 × 10^9^ CFU/ml and min is 6.48 × 10^9^ CFU/ml. (**D**) *C. sakazakii* at -7 log dilution; Avg is 6.8 × 10^9^ CFU/ml, max is 8.0 × 10^9^ CFU/ml and min is 5.6 × 10^9^ CFU/ml.

To establish baseline bacterial concentrations for subsequent molecular detection comparisons, colonies of *S. enterica* and *C. sakazakii* were enumerated at different dilutions when cultures were standardized to OD_600_ = 1.0 (Fig. 2). For *S. enterica*, the average colony count were 7.72 ± 1.36 × 10^9^ CFU/ml (6.28 - 8.92 × 10^9^ CFU/ml) and 8.4 ± 1.6 × 10^9^ CFU/ml (6.0 - 10.8 × 10^9^ CFU/ml) at dilution factors of 10^-6^ and 10^-7^, respectively. For *C. sakazakii*, the average colony counts were 7.08 ± 0.52 × 10^9^ CFU/ml (6.48 - 7.68 × 10^9^ CFU/ml) and 6.8 ± 1.2 × 10^9^ CFU/ml (5.6 - 8.0 × 10^9^ CFU/ml) at dilution factors of 10^-6^ and 10^-7^, respectively.

### Inhibition of gDNA Amplification by Direct PMA Treatment

To confirm the covalent binding between PMA and bacterial gDNA extracted from both species, gDNA was directly treated with 100 μM of PMA under various conditions. All gDNA was categorized as PMA-treated (+) or PMA-untreated (-) for *S. enterica* and *C. sakazakii* live and dead bacteria, respectively (Table 2). For gDNA (- PMA), the Ct values for *S. enterica* and *C. sakazakii* were found to be 13 and 24, respectively. In contrast, the gDNA (+ PMA) of both bacteria showed significantly higher Ct values (> 30), indicating the inhibition effect of PMA for had an inhibition effect on DNA amplification. This inhibition was consistent across all PMA-treated samples, regardless of the bacterial species.

### Selection of optimal OD_600_ Concentration for Bacterial Experiments

**Fig. 3:**
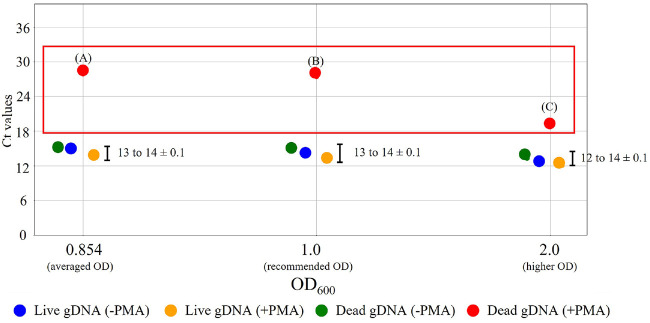
Comparison of Ct values across different OD_600_ values for *S. enterica*. Three different OD600 were shown with Ct values from averaged OD600 (0.854), recommended OD600 (1.0), and higher OD600 (2.0). (A) = 28 ± 0.4, (B) = 28 ± 0.1, (C) = 18 ± 0.3.

To establish optimal bacterial concentrations for consistent v-PCR discrimination between live and dead bacteria, three different OD_600_ conditions were systematically evaluated. This optimization was essential to ensure reproducible PMA binding and prevent incomplete inhibition of dead bacterial DNA amplification. The three OD_600_ conditions tested included: (i) an average OD_600_ of 0.854, representing the mean value derived from ten-fold diluted initial bacterial culture measurements; (ii) a recommended OD_600_ of 1.0, based on standard protocols from previous viability-PCR studies [30, 40]; and (iii) a higher OD_600_ of 2.0, included for a comparative analysis to assess potential concentration-dependent effects on PMA efficiency. The 0.854 and 1.0 OD_600_ conditions showed similar results, particularly for the Ct values of dead gDNA (+ PMA) (Fig. 3). In contrast, higher OD_600_ conditions resulted in lower Ct values for dead gDNA (+ PMA), likely due to incomplete PMA binding to residual gDNA [41]. Based on these results and previous studies, an OD_600_ of 1.0 was selected as the optimal condition for subsequent experiments. This standardization enhances the experimental reproducibility and facilitates reliable cross-study comparisons.

### Optimization of PMA Concentration for Effective Discrimination between Live and Dead Bacteria

**Fig. 4:**
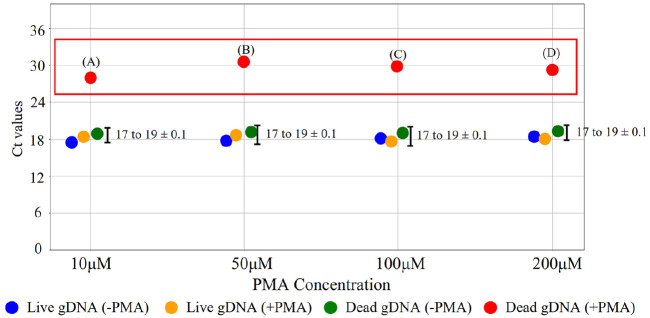
Ct values for different PMA concentrations in *S. enterica*. Four different PMA concentrations were evaluated to compare Ct values. (A) 10 μM of PMA (28 ± 0.1), (B) 50 μM of PMA (31 ± 0.1), (C) 100 μM of PMA (30 ± 0.1), (D) 200 μM of PMA (29 ± 0.1).

To determine the optimal PMA concentration, Ct values were compared across four PMA concentrations (10, 50, 100, and 200 μM) under four bacterial conditions (Fig. 1). In the red box, the results showed elevated Ct values (≥30) in dead gDNA (+PMA) samples compared with the other conditions (Fig. 4). At concentrations of 50 and 100 μM, Ct values exceeded 30, indicating effective inhibition of DNA amplification in dead bacteria. In contrast, 10 μM PMA showed insufficient inhibition owing to its low concentration. In contrast, at 200 μM, the Ct values slightly decreased, indicating that an excessively high concentration might reduce its inhibition effectiveness. The other three conditions, live gDNA (± PMA) and dead gDNA (− PMA), maintained consistent Ct values between 17 and 19, indicating a minimal effect of PMA on live bacteria. At a concentration of 100 μM, the PCR amplification of live bacteria increased slightly, while effectively inhibited dead bacterial DNA. This enhanced the effectiveness of this concentration and improved the discrimination between live and the PMA dead bacteria. The inhibition patterns of 50 and 100 μM PMA were similar. However, 100 μM was selected as the optimal concentration because of its improved experimental stability, consistent with previous studies, and it also showed more pronounced differences in PCR amplification between PMA-treated dead bacteria and treated live bacteria [26, 42]. On the basis of these advantages, combined with the standard deviations (SD) maintained below 0.1, 100 μM was established as the most suitable concentration. Statistical analysis confirmed that all conditions showed highly significant differences compared to dead gDNA (+PMA) across all concentrations tested (*p* < 0.001 for all comparisons; Supplementary Data S1).

### Optimization of Triton X-100 Concentration for Enhanced PMA Efficiency

**Fig. 5:**
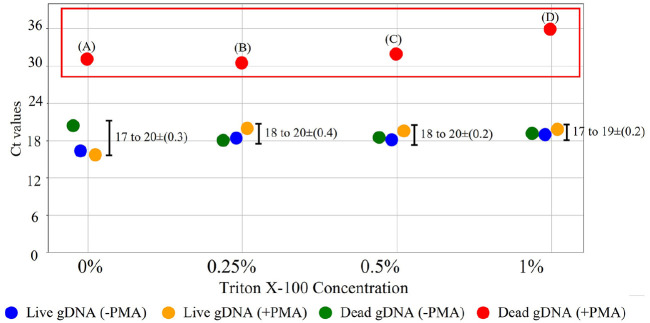
Effect of Triton X-100 concentration on PCR amplification efficiency in the PMA-treated samples. Four different Triton X-100 concentrations were evaluated to compare Ct values. (A) = 31 ± 0.4, (B) = 30 ± 0.4, (C) = 32 ± 0.6, (D) = 36 ± 0.3.

The effect of varying the Triton X-100 concentration (0%, 0.25%, 0.5%, and 1%) was evaluated with the aim of enhancing PMA efficiency, increasing membrane penetration in dead bacteria, and minimizing the effects on live bacteria (Fig. 5). In live bacteria, Ct values remained stable at 18 across all Triton X-100 concentrations, regardless of PMA treatment, indicating that Triton X-100 had a minimal impact on live gDNA. Dead gDNA (+ PMA) demonstrated significantly higher Ct values, ranging from 30 to 36, which rose with increasing Triton X-100 concentration. Notably, at 1% Triton X-100, the Ct values reached a maximum of 36, indicating optimal PMA binding efficiency. The increasing Ct values at higher Triton X-100 concentrations indicate that PMA penetration and binding to dead gDNA increased, effectively inhibiting gDNA amplification. All the experimental results showed acceptable reproducibility, with standard deviations below 0.4. Based on these results, 1% Triton X-100 demonstrated the highest Ct value of 36 for dead gDNA (+ PMA) and maintained a stable Ct value of 18 for live bacteria, indicating it was the optimal concentration. Statistical analysis demonstrated highly significant differences between all conditions and dead gDNA (+PMA) at each Triton X-100 concentration (*p* < 0.001 for all comparisons; Supplementary Data S1).

### Comparative Analysis of CFU Counts and ddPCR Copy Numbers

**Fig. 6:**
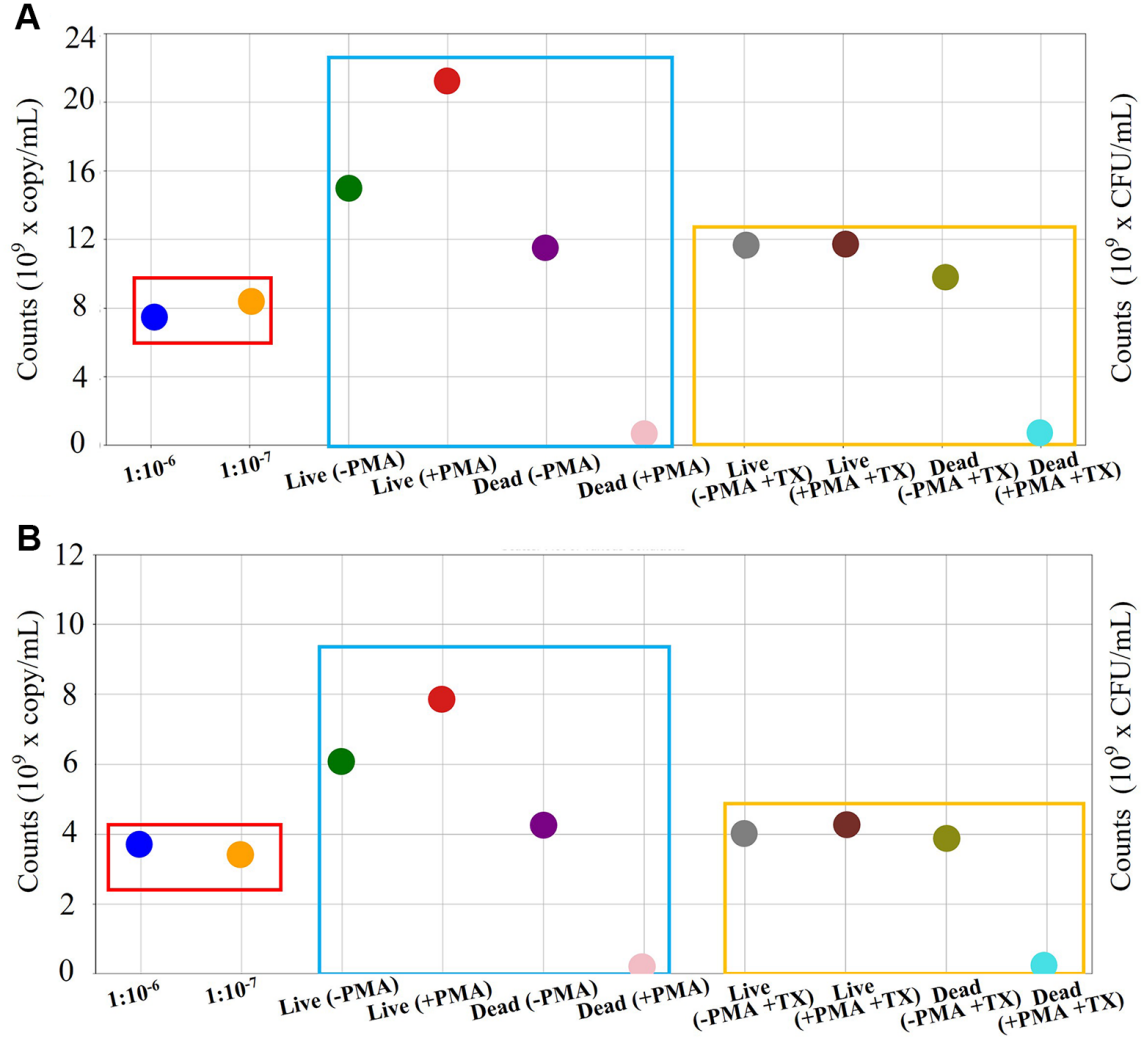
CFU and copy numbers of *S. enterica* (A) and *C. sakazakii* (B). The red box represents CFU counts, the blue box shows ddPCR copy numbers without Triton X-100, and the yellow box shows ddPCR copy numbers with Triton X-100. The minus (-) and plus (+) symbols represent untreated and treated conditions, respectively.

The optimized conditions, specifically OD_600_ 1.0, PMA 100 μM, and Triton X-100 concentrations of 0% and 1%, established by qPCR, were validated through ddPCR and CFU counts for both *S. enterica* and *C. sakazakii* (Fig. 6). Although copy numbers differed between the species, both showed similar patterns. At 0% Triton X-100, live gDNA (+ PMA) exhibited the highest copy numbers with *S. enterica* (21 × 10^9^ copies/ml) and *C. sakazakii* (15 × 10^9^ copies/ml), whereas live gDNA (- PMA) exhibited 15 × 10^9^ copies/ml for *S. enterica* and 12 × 10^9^ copies/ml for *C. sakazakii* (Supplementary Data S2). CFU counts were lower than ddPCR copy numbers, ranging from 7.72 to 8.4 × 10^9^ CFU/ml for *S. enterica* and 6.8 to 7.1 × 10^9^ CFU/ml for *C. sakazakii*. At 1% Triton X-100, the copy numbers decreased significantly under both live conditions, to approximately 11 × 10^9^ copies/ml for *S. enterica* and 8 × 10^9^ copies/ml for *C. sakazakii*. Dead gDNA (+PMA) showed no detectable amplification, regardless of the Triton X-100 concentration. These patterns were consistent across both species, demonstrating that dead gDNA amplification by PMA in combination with Triton X-100 was effectively inhibited, although the higher ddPCR copy numbers compared to the CFU counts warrant further investigation.

## Discussion

Both qPCR and ddPCR effectively inhibited dead gDNA amplification with PMA treatment. Although ddPCR showed complete inhibition with zero copy numbers, qPCR results with Ct values above 30 suggested an amplification potential (Figs. 5 and 6). Statistical analysis confirmed highly significant differences (*p* < 0.001) between dead gDNA (+PMA) and all other conditions across both PMA and Triton X-100 concentrations, demonstrating the robust discriminatory power of the optimized v-PCR protocol. The detection of residual signals in qPCR (Ct > 30) despite PMA treatment can be attributed to its relative quantification, where Ct values above 36 cannot definitively confirm zero detection of bacteria [43]. In this experiments, no DNA control samples consistently showed amplification within 40 cycles. This indicated that the observed signals were specific to bacterial DNA rather than contamination or non-specific amplification. However, ddPCR copy number quantification provided absolute confirmation when no copy was detected. These results demonstrated the superior accuracy of ddPCR absolute quantification [18, 21], whereas qPCR offers advantages in experimental compatibility and ease of use [43]. In addition, qPCR provides rapid and broad applicability under various experimental conditions. As recommended by the MFDS, qPCR is required for the initial detection of foodborne pathogens [6]. On the other hand, ddPCR offers precise quantification [13, 17], although it requires complex experimental processes. Therefore, both methods were complementary in this study. The combination of these methods ensures practical utility and analytical accuracy of pathogen detection.

A hypothesis to explain the observed differences in copy number between TX-treated and untreated live gDNA samples (Fig. 6) was subsequently evaluated. External bias is known to influence PCR amplification through salt contamination and the formation of a GC-rich secondary structure [44]. While the exact underlying mechanism remains unclear, we hypothesized that PMA treatment may alter the accessibility or conformation of DNA templates such that they potentially would be more suitable for primer binding and polymerase access. However, the proposed mechanism requires further investigation through detailed biochemical studies to establish the underlying molecular interactions. Based on these results, the most plausible hypothesis was that PMA enhances PCR amplification. Additionally, this study used a single representative strain for each species, which may limit the generalizability of the findings across different strains that could vary in membrane characteristics and surfactant sensitivity. Future studies should evaluate the method across multiple strains to confirm its broader applicability and establish strain-independent optimization parameters.

Several studies have explored v-PCR approaches using PMA and surfactants for the detection of foodborne pathogens. For *Staphylococcus aureus*, researchers have developed a PMA-qPCR assay to distinguish between live and dead cells, but this approach lacks enhancement with a surfactant and absolute quantification capabilities [45]. Similarly, PMA-based v-PCR protocols have enhanced *Salmonella* Enteritidis detection [46], while lactic acid enhancement improved *E. coli* viability detection [47], and PMA-coupled assays have enabled *Xanthomonas* quantification in agricultural applications [48]. Most relevant to this study, Triton X-100 combined with PMA enhanced the detection of viable *Campylobacter* and *Salmonella* in qPCR assays [49]. However, this study provides practical optimization of the combined v-ddPCR approach through three key contributions. First, we systematically evaluated PMA and Triton X-100 concentrations to enhance viability discrimination between live and dead bacteria. Second, we demonstrated ddPCR's superior quantitative capabilities over qPCR for definitive detection of dead bacteria. Third, we validated this optimized protocol across two important foodborne pathogens and achieved improved correlation with traditional CFU enumeration. This enhanced correlation addresses quantitative discrepancies commonly observed in molecular viability approaches and thereby improves the method's practical utility for food safety applications. The proposed integration of ddPCR provides definitive confirmation that zero detection confirms the complete inhibition of dead bacterial DNA amplification, superior to relative Ct value assessments. Nevertheless, a significant limitation of this study is that the evaluation was conducted solely with laboratory-cultured bacterial isolates rather than real or spiked food matrices. This limits the direct applicability of this findings to practical food safety scenarios and represents an important area for future validation studies.

In this study, we aimed to evaluate differences between CFU counts and ddPCR copy numbers for bacterial quantification. Owing to the inherent unit differences between these measurements (CFU/ml and copies/ml), a direct comparison was not possible [28, 50]. Therefore, comparisons were based on back calculations of the initial bacterial broth culture to provide insights into methodological differences (Supplementary Data S2). Several hypotheses have been proposed to explain these differences. First, the fundamental difference between the CFU counts and ddPCR copy numbers results from their distinct measurement targets [51, 52]. CFU counts measure only viable and cultivable bacteria, whereas ddPCR quantifies total gDNA, including both live and dead bacteria [51-53]. potentially contributes to the discrepancies in their results [50-52]. Significant differences were observed in the quantification. When ddPCR was adjusted to an OD_600_ of the 1.0, CFU count was not adjusted [34]. To address this difference, ddPCR copy numbers were back-calculated by multiplying with the diluted broth factor of OD_600_. However, these numbers did not match the CFU counts. This could be due to the variability in both the gDNA extraction process from cultured bacteria and the cultivation process of live bacteria and this inaccuracy might indicate differences in copy numbers [40, 54, 55]. However, these differences were reduced by treatment with Triton X-100 (Supplementary Data S2). Although the exact mechanism requires further investigation, the data showed that Triton X-100 treatment resulted in copy numbers that were more closely aligned with CFU counts, suggesting improved quantification accuracy [35, 56]. Unit differences and the quantification process are as particularly significant because of their fundamental impact on copy number estimation. This appears to be the most crucial factor among the hypotheses presented, with standardization and quantification issues being central to resolving these discrepancies.

The observed baseline Ct value variations between experiments (Figs. 3-5) can be attributed to several methodological and biological factors inherent to the viability of PCR approaches. First, cellular physiological states may vary, even within uniform bacterial cultures and affect membrane permeability and PMA efficiency. As demonstrated in viability dye studies, membrane permeability changes in live cells depending on the physiological status, with rapidly dividing and senescent cells being most affected by dye treatment due to cell wall composition changes throughout different growth phases [42]. Second, qPCR methodology inherently includes inter- and intra-assay variations that can influence baseline quantification, as PCR-based methods are sensitive to minor differences in experimental conditions [43]. Importantly, despite these baseline variations, the core principle of our v-PCR approach was consistent across all experiments. PMA treatment significantly increased the Ct values in dead bacteria, demonstrating effective covalent binding to dead bacterial DNA and subsequent amplification inhibition. The relative differences between live and dead bacteria remained substantial and statistically significant under all experimental conditions, thus supporting the reliability and selectivity of our optimized v-PCR protocol.

This study demonstrates the potential of v-PCR as a promising alternative to conventional CFU counting, which has long been the standard method for microbial quantification. This optimized v-ddPCR with Triton X-100 showed higher detection sensitivity than the conventional culture methods (Fig. 6). This difference in quantification raises an important consideration regarding Viable But Non-Culturable (VBNC) bacterial states, which present a significant challenge in food safety monitoring. Although this study did not specifically target VBNC populations, the methodology developed provides a foundation for future applications in this area. Bacteria in the VBNC state maintain metabolic activity and potentially retain infectivity despite being unable to grow in standard culture media, often leading to false-negative results when using culture-based detection methods [57, 58]. The v-ddPCR approach with PMA treatment has theoretical advantages in detecting such populations, because it targets membrane integrity rather than cultivability [59]. Although PCR-based methods face certain limitations in the accurate quantification of VBNC [60], incorporating of Triton X-100 provides a key advance in the v-PCR methodology. The results demonstrate that this modification improves the discrimination between live and dead bacteria (Fig. 5), although the specific mechanism by which Triton X-100 enhances PMA selectivity requires further mechanistic investigation. Future studies specifically investigating the application of this optimized protocol to VBNC bacterial populations in food matrices would be valuable for establishing its utility in food safety systems.

## Conclusion

By integrating v-PCR with Triton X-100, this study enhanced bacterial detection and effectively addressed a critical challenge in food safety systems: the trace amounts of live bacteria. The combination of the rapid screening capability of qPCR and precise quantification of ddPCR offers a practical solution that was developed in alignment with the MFDS guidelines. However, the generalizability of these findings is limited by the testing of only two bacterial strains (*S. enterica* and *C. sakazakii*). Furthermore, the mechanism by which Triton X-100 enhances PMA selectivity remains speculative and requires further investigation. The strategic use of Triton X-100 showed improved sensitivity for pathogen detection in the tested species, minimizing false-negative results compared with conventional culture-based techniques. Although this method shows promise for enhancing food safety through improved microbiological monitoring, validation across diverse bacterial species and food matrices is essential before widespread implementation. With appropriate validation, this approach could effectively prevent foodborne illnesses by reducing risk, ultimately contributing to enhanced public health protection and food safety management.

## Supplemental Materials

Supplementary data for this paper are available on-line only at http://jmb.or.kr.



## Figures and Tables

**Table 1:** Specifications of primer and probe sets for foodborne pathogens.

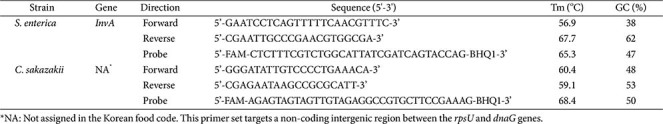

**Table 2:** Ct values from direct PMA treatment of bacterial gDNA.

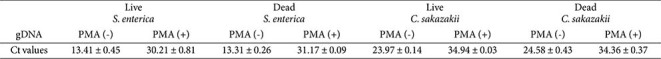

## References

[1] Effland T, Lawson A, Balter S, Devinney K, Reddy V, Waechter H (2018). Discovering foodborne illness in online restaurant reviews. J. Am. Med. Inform. Assoc..

[2] Lee JK, Kwak NS, Kim HJ (2016). Systemic analysis of foodborne disease outbreak in Korea. Foodborne Pathog. Dis..

[3] Bintsis T (2017). Foodborne pathogens. AIMS Microbiol..

[4] Ministry of Food and Drug Safety. 2025. Statistics on foodborne illness outbreaks in Korea. Available from https://www.index.go.kr/unity/potal/main/EachDtlPageDetail.do?idx_cd=2761. Accessed Aug. 5, 2025.

[5] Kim DH, Jeong D, Song KY, Kang IB, Kim H, Seo KH (2018). Culture supernatant produced by *Lactobacillus kefiri* from kefir inhibits the growth of *Cronobacter sakazakii*. J. Dairy Res..

[6] Kim SO, Kim SS (2021). Bacterial pathogen detection by conventional culture-based and recent alternative (polymerase chain reaction, isothermal amplification, enzyme linked immunosorbent assay, bacteriophage amplification, and gold nanoparticle aggregation) methods in food samples: a review. J. Food Saf..

[7] Andino A, Hanning I (2015). *Salmonella enterica*: survival, colonization, and virulence differences among serovars. ScientificWorldJournal.

[8] Eng SK, Pusparajah P, Ab Mutalib NS, Ser HL, Chan KG, Lee LH (2015). *Salmonella*: a review on pathogenesis, epidemiology and antibiotic resistance. Front. Life Sci..

[9] Singh N, Goel G, Raghav M (2015). Insights into virulence factors determining the pathogenicity of *Cronobacter sakazakii*. Virulence.

[10] Wattiau P, Boland C, Bertrand S (2011). Methodologies for *Salmonella enterica* subsp. enterica subtyping: gold standards and alternatives. Appl. Environ. Microbiol..

[11] Mazi IM, Onyeaka H, Nnaji ND (2023). Foodborne pathogens in Africa: understanding *Cronobacter sakazakii*. Public Health Chall..

[12] Hu L, Fu Y, Zhang S, Pan Z, Xia J, Zhu P (2022). An assay combining droplet digital PCR with propidium monoazide treatment for the accurate detection of live cells of *Vibrio vulnificus* in plasma samples. Front. Microbiol..

[13] Klein D (2002). Quantification using real-time PCR technology: applications and limitations. Trends Mol. Med..

[14] Persson S, Eriksson R, Lowther J, Ellström P, Simonsson M (2018). Comparison between RT droplet digital PCR and RT real-time PCR for quantification of noroviruses in oysters. Int. J. Food Microbiol..

[15] Zhao Y, Xia Q, Yin Y, Wang Z (2016). Comparison of droplet digital PCR and quantitative PCR assays for quantitative detection of *Xanthomonas citri* subsp. Citri. PLoS One.

[16] Harshitha R, Arunraj DR (2021). Real-time quantitative PCR: a tool for absolute and relative quantification. Biochem. Mol. Biol. Educ..

[17] Mackay IM (2004). Real-time PCR in the microbiology laboratory. Clin. Microbiol. Infect..

[18] Hindson BJ, Ness KD, Masquelier DA, Belgrader P, Heredia NJ, Makarewicz AJ (2011). High-throughput droplet digital PCR system for absolute quantitation of DNA copy number. Anal. Chem..

[19] Salipante SJ, Jerome KR (2020). Digital PCR-an emerging technology with broad applications in microbiology. Clin. Chem..

[20] Park C, Park D, Hassan ZU, Choi SH, Kim S (2023). Comparison of RT-qPCR and RT-ddPCR with Rift valley fever virus (RVFV) RNA. Sci. Rep..

[21] Choi CH, Kim E, Yang SM, Kim DS, Suh SM, Lee GY (2022). Comparison of real-time PCR and droplet digital PCR for the quantitative detection of *Lactiplantibacillus plantarum* subsp. plantarum. Foods.

[22] Hindson CM, Chevillet JR, Briggs HA, Gallichotte EN, Ruf IK, Hindson BJ (2013). Absolute quantification by droplet digital PCR versus analog real-time PCR. Nat. Methods.

[23] Daddy Gaoh S, Kweon O, Lee YJ, Hussong D, Marasa B, Ahn Y (2022). A propidium monoazide (PMAxx)-droplet digital PCR (ddPCR) for the detection of viable *Burkholderia cepacia* complex in nuclease-free water and antiseptics. Microorganisms.

[24] Park C, Park J, Chang D, Kim S (2025). Development of reference-based model for improved analysis of bacterial community. Food Res. Int..

[25] Pinheiro LB, Coleman VA, Hindson CM, Herrmann J, Hindson BJ, Bhat S (2012). Evaluation of a droplet digital polymerase chain reaction format for DNA copy number quantification. Anal. Chem..

[26] Gobert G, Cotillard A, Fourmestraux C, Pruvost L, Miguet J, Boyer M (2018). Droplet digital PCR improves absolute quantification of viable lactic acid bacteria in faecal samples. J. Microbiol. Methods.

[27] Wouters Y, Dalloyaux D, Christenhusz A, Roelofs HMJ, Wertheim HF, Bleeker-Rovers CP (2020). Droplet digital polymerase chain reaction for rapid broad-spectrum detection of bloodstream infections. Microb. Biotechnol..

[28] Lee JL, Levin RE (2009). Discrimination of viable and dead *Vibrio vulnificus* after refrigerated and frozen storage using EMA, sodium deoxycholate and real-time PCR. J. Microbiol. Methods.

[29] Zhao S, Zhang J, Li Z, Han Y, Kan B (2021). Enumeration of viable non-culturable *Vibrio cholerae* using droplet digital PCR combined with propidium monoazide treatment. Front. Cell. Infect. Microbiol..

[30] Nkuipou-Kenfack E, Engel H, Fakih S, Nocker A (2013). Improving efficiency of viability-PCR for selective detection of live cells. J. Microbiol. Methods.

[31] Seinige D, Krischek C, Klein G, Kehrenberg C (2014). Comparative analysis and limitations of ethidium monoazide and propidium monoazide treatments for the differentiation of viable and nonviable Campylobacter cells. Appl. Environ. Microbiol..

[32] Koley D, Bard AJ (2010). Triton X-100 concentration effects on membrane permeability of a single HeLa cell by scanning electrochemical microscopy (SECM). Proc. Natl. Acad. Sci. USA.

[33] Kalam S, Abu-Khamsin SA, Kamal MS, Patil S (2021). Surfactant adsorption isotherms: a review. ACS Omega.

[34] Nocker A, Mazza A, Masson L, Camper AK, Brousseau R (2009). Selective detection of live bacteria combining propidium monoazide sample treatment with microarray technology. J. Microbiol. Methods.

[35] Tan SW, Gooran N, Lim HM, Yoon BK, Jackman JA (2023). Tethered bilayer lipid membrane platform for screening triton X-100 detergent replacements by electrochemical impedance spectroscopy. Nanomaterials.

[36] Abu-Ghunmi L, Badawi M, Fayyad M (2014). Fate of triton X-100 applications on water and soil environments: a review. J. Surfactants Deterg..

[37] Ministry of Food and Drug Safety. 2024. Foodsafetykorea: Foodcode. Available from https://various.foodsafetykorea.go.kr/fsd/#/ext/Document/FC. Accessed Aug. 5, 2025.

[38] Nocker A, Sossa-fernandez P, Burr MD, Camper AK (2007). Use of propidium monoazide for live/ dead distinction in microbial ecology. Appl. Environ. Microbiol..

[39] Nocker A, Cheung CY, Camper AK (2006). Comparison of propidium monoazide with ethidium monoazide for differentiation of live vs. dead bacteria by selective removal of DNA from dead cells. J. Microbiol. Methods.

[40] Dionisi HM, Harms G, Layton AC, Gregory IR, Parker J, Hawkins SA (2003). Power analysis for real-time PCR quantification of genes in activated sludge and analysis of the variability introduced by DNA extraction. Appl. Environ. Microbiol..

[41] Kaur S, Bran L, Rudakov G, Wang J, Verma MS (2025). Propidium monoazide is unreliable for quantitative live-dead molecular assays. Anal. Chem..

[42] Fittipaldi M, Nocker A, Codony F (2012). Progress in understanding preferential detection of live cells using viability dyes in combination with DNA amplification. J. Microbiol. Methods.

[43] Pfaffl MW (2001). A new mathematical model for relative quantification in real-time RT-PCR. Nucleic Acids Res..

[44] Mutter GL, Boynton KA (1995). PCR bias in amplification of androgen receptor alleles, a trinucleotide repeat marker used in clonality studies. Nucleic Acids Res..

[45] Zi C, Zeng D, Ling N, Dai J, Xue F, Jiang Y (2018). An improved assay for rapid detection of viable *Staphylococcus aureus* cells by incorporating surfactant and PMA treatments in qPCR. BMC Microbiol..

[46] Thilakarathna SH, Stokowski T, Chui L (2022). An improved real-time viability PCR assay to detect *Salmonella* in a culture-independent era. Int. J. Mol. Sci..

[47] Dinu LD, Al-Zaidi QJ, Matache AG, Matei F (2024). Improving the efficiency of viability-qPCR with lactic acid enhancer for the selective detection of live pathogens in foods. Foods.

[48] Wang H, Wagnon R, Moreno D, Timilsina S, Jones J, Vallad G (2022). A Long-amplicon viability-qPCR test for quantifying living pathogens that cause bacterial spot in tomato seed. Plant Dis..

[49] Liu H, Meng F, Nyaruaba R, He P, Hong W, Jiang M (2022). A triton X-100 assisted PMAxx-qPCR assay for rapid assessment of infectious African swine fever virus. Front. Microbiol..

[50] Papić B, Pate M, Henigman U, Zajc U, Gruntar I, Biasizzo M (2017). New approaches on quantification of *Campylobacter jejuni* in poultry samples: the use of digital PCR and real-time PCR against the ISO standard plate count method. Front. Microbiol..

[51] Kiefer A, Tang P, Arndt S, Fallico V, Wong C (2020). Optimization of viability treatment essential for accurate droplet digital PCR enumeration of probiotics. Front. Microbiol..

[52] Santander RD, Meredith CL, Aćimović SG (2019). Development of a viability digital PCR protocol for the selective detection and quantification of live *Erwinia amylovora* cells in cankers. Sci. Rep..

[53] Davis C (2014). Enumeration of probiotic strains: Review of culture-dependent and alternative techniques to quantify viable bacteria. J. Microbiol. Methods.

[54] Cankar K, Stebih D, Dreo T, Žel J, Gruden K (2006). Critical points of DNA quantification by real-time PCR - effects of DNA extraction method and sample matrix on quantification of genetically modified organisms. BMC Biotechnol..

[55] Wagner AO, Praeg N, Reitschuler C, Illmer P (2015). Effect of DNA extraction procedure, repeated extraction and ethidium monoazide (EMA)/propidium monoazide (PMA) treatment on overall DNA yield and impact on microbial fingerprints for bacteria, fungi and archaea in a reference soil. Appl. Soil Ecol..

[56] Remy MM, Alfter M, Chiem MN, Barbani MT, Engler OB, Suter-Riniker F (2019). Effective chemical virus inactivation of patient serum compatible with accurate serodiagnosis of infections. Clin. Microbiol. Infect..

[57] Trevors JT, van Elsas JD, Bej AK. 2013. The molecularly crowded cytoplasm of bacterial cells: dividing cells contrasted with viable but non-culturable (VBNC) bacterial cells. *Curr. Issues Mol. Biol.* **15:** 1-6. PMID: 22513407. 22513407

[58] Wideman NE, Oliver JD, Crandall PG, Jarvis NA (2021). Detection and potential virulence of viable but non-culturable (VBNC) listeria monocytogenes: a review. Microorganisms.

[59] Rowlands V, Rutkowski AJ, Meuser E, Carr TH, Harrington EA, Barrett JC (2019). Optimisation of robust singleplex and multiplex droplet digital PCR assays for high confidence mutation detection in circulating tumour DNA. Sci. Rep..

[60] Okada A, Tsuchida M, Rahman MM, Inoshima Y (2022). Two-round treatment with propidium monoazide completely inhibits the detection of dead *Campylobacter* spp. cells by quantitative PCR. Front. Microbiol..

